# Accumulation of Polyunsaturated Aldehydes in the Gonads of the Copepod *Acartia tonsa* Revealed by Tailored Fluorescent Probes

**DOI:** 10.1371/journal.pone.0112522

**Published:** 2014-11-10

**Authors:** Stefanie Wolfram, Jens C. Nejstgaard, Georg Pohnert

**Affiliations:** 1 Institute for Inorganic and Analytical Chemistry, Friedrich Schiller University, Jena, Germany; 2 Skidaway Institute of Oceanography, Savannah, GA, United States of America; 3 Department of Experimental Limnology, Leibniz-Institute of Freshwater Ecology and Inland Fisheries (IGB), Department 3 Experimental Limnology, Stechlin, Germany; Inserm, France

## Abstract

Polyunsaturated aldehydes (PUAs) are released by several diatom species during predation. Besides other attributed activities, these oxylipins can interfere with the reproduction of copepods, important predators of diatoms. While intensive research has been carried out to document the effects of PUAs on copepod reproduction, little is known about the underlying mechanistic aspects of PUA action. Especially PUA uptake and accumulation in copepods has not been addressed to date. To investigate how PUAs are taken up and interfere with the reproduction in copepods we developed a fluorescent probe containing the α,β,γ,δ-unsaturated aldehyde structure element that is essential for the activity of PUAs as well as a set of control probes. We developed incubation and monitoring procedures for adult females of the calanoid copepod *Acartia tonsa* and show that the PUA derived fluorescent molecular probe selectively accumulates in the gonads of this copepod. In contrast, a saturated aldehyde derived probe of an inactive parent molecule was enriched in the lipid sac. This leads to a model for PUAs' teratogenic mode of action involving accumulation and covalent interaction with nucleophilic moieties in the copepod reproductive tissue. The teratogenic effect of PUAs can therefore be explained by a selective targeting of the molecules into the reproductive tissue of the herbivores, while more lipophilic but otherwise strongly related structures end up in lipid bodies.

## Introduction

Diatoms are highly abundant marine phytoplankton and considered to be among the most important food sources for the dominating zooplankton such as copepods. Diatoms are thus at the bottom of the marine food web and also play central roles in climate functioning [1]. The beneficial role of diatoms as food source was however questioned due to evidence of decreased reproductive success in copepods feeding on diatom rich diets, compared to various non-diatom prey types (reviewed in [2], [3]). Since these initial studies the effect of diatoms on copepod reproduction has been intensively investigated in field and laboratory experiments (reviewed in [4]–[6]). The production of teratogenic α,β,γ,δ-polyunsaturated aldehydes (PUAs) was made responsible for the reduced hatching success of copepod eggs and apoptotic malformations of copepod offspring [5], [7], [8]. While PUAs can explain several instances of poor copepod reproduction, their negative impact is not universal. Several field and laboratory assays gave no evidence of adverse PUA effects [9]–[13] while others did (reviewed in [5]). Higher molecular weight oxylipins and other toxins might provide additional explanations for observed teratogenic effects of diatoms on copepods [4], [5]. Nutritional inadequacies of some diatom species [14] and poor nutrient uptake due to rapid gut passage time [15] were also discussed. Most reports fuelling the debate about PUA toxicity focus on observations of the outcome of feeding experiments, while very few mechanistic studies address the origin of these findings and the mode of action of PUAs [9], [16]. Data is especially lacking on how the metabolites are delivered to and distributed in feeding copepods.

Production of PUAs is initiated when diatom cells are wounded in the feeding organs of the herbivores and is even observed in the guts of the animals [17], [18]. Thus proper experiments on uptake and targeting of PUAs would have to involve an active feeding process for delivery. Few PUA carriers have been introduced for feeding experiments that mimic the situation in the plankton. Buttino et al. [19] delivered the PUA 2,4-decadienal (DD) encapsulated in giant liposomes and observed reduced egg hatching success as well as induction of apoptosis in female tissue and copepod embryos of *Temora stylifera* and *Calanus helgolandicus*. Ianora et al. [7] incubated the non PUA-producing dinoflagellate *Prorocentrum minimum* as neutral living carrier with DD that was then delivered to copepods in feeding experiments. But no feeding protocols using living food sources that allow detection of fluorescently labeled probes without interfering photosynthetic pigments have been available to date. This has hampered a mechanistic evaluation of PUA delivery in the animals.

Here we introduce a feeding protocol that allows delivery of fluorescent probes using the heterotrophic dinoflagellate *Oxyrrhis marina* as a vector. We use this procedure to deliver novel fluorescent molecular probes to adults of *Acartia tonsa*. This calanoid copepod is commonly found in coastal waters in a wide geographical range including the Antlantic, Indian and Pacific Oceans, the Baltic, Mediterranean and North Seas [20], [21]. For *Acartia spp*. first experiments on the effects of diatoms in general as well as PUA producers were performed. No teratogenic effect was observed with *A. tonsa* feeding on a diatom rich natural diet but no information about the PUA content of this mixed diet was reported [22]. In specific feeding experiments the PUA producer *Thalassiosira rotula* caused reduced egg production and hatching success and led to blockage of egg development in *Acartia clausi* [23].

**Figure 1:**
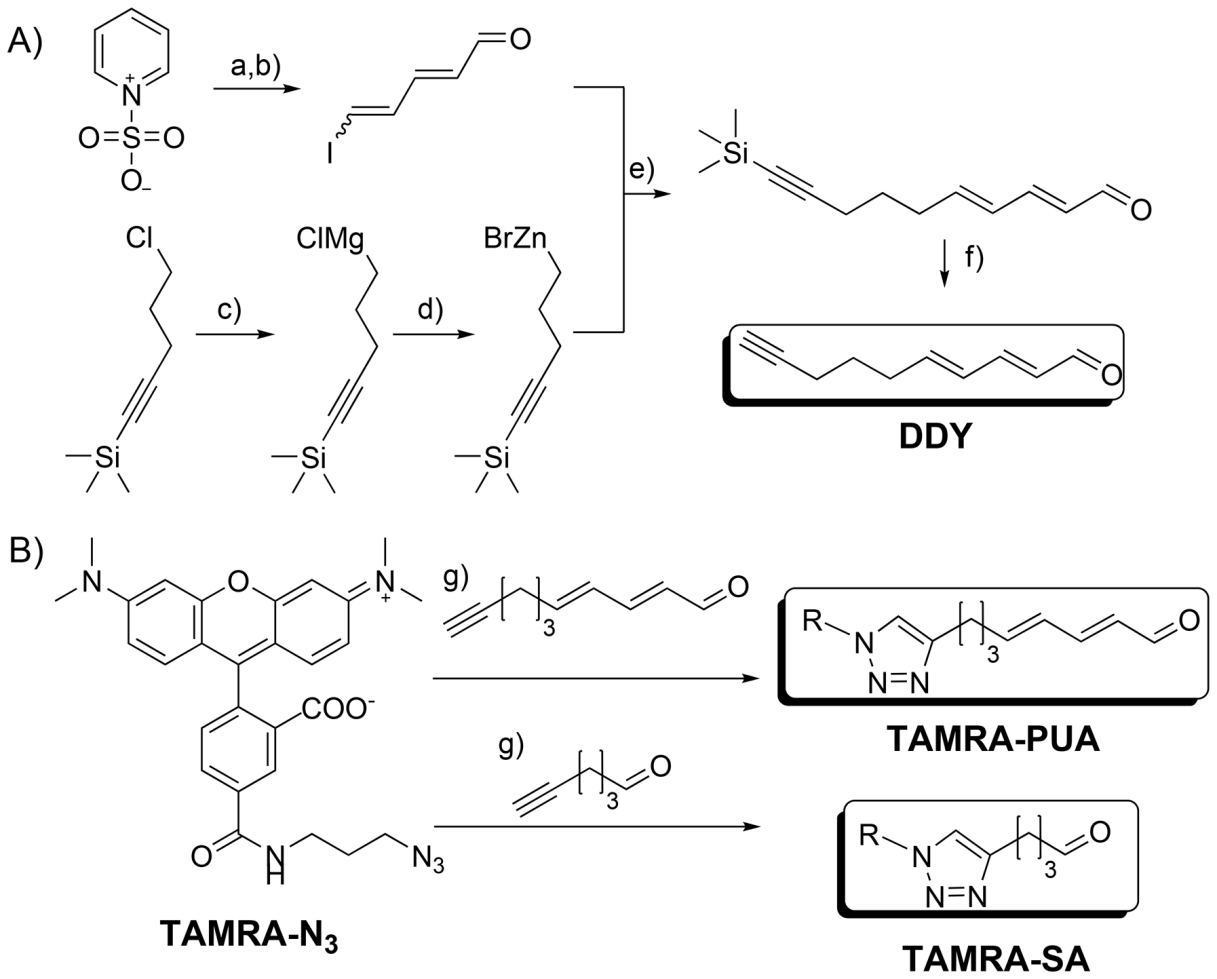
Synthesis of TAMRA-PUA and TARMA-SA. A) Synthesis of DDY, conditions: a) KOH, H_2_O, 22% yield; b) 1.2 equ. PPh_3_, 1.2 equ. I_2_, CH_2_Cl_2_, 55% yield; c) 3.2 equ. Mg, C_2_H_4_Br_2_, THF, d) 1.2 equ. ZnBr_2_, THF; e) 0.06 equ. Pd(PPh_3_)_4_, THF, 28 to 30% yield; f) 1.2 equ. TBAF, THF, H_2_O, 30% yield; B) Synthesis of the probes, conditions: g) tris[(1-benzyl-1*H*-1,2,3-triazol-4-yl)methyl]amine), sodium ascorbate, copper sulfate, 68% and 77% yield. R = 5-N-propylcarbamoyl tetramethylrhodamine. For detailed experimental procedures and product characterization see Information S1.

The applied probe for monitoring the targeting of PUA consists of a head group containing the α,β,γ,δ-unsaturated aldehyde motive and a tetramethylrhodamine based fluorescent reporter (TAMRA-PUA in Figure 1). The general layout follows concepts of activity-based protein profiling that allow delivery of nucleophilic target molecules to biological matrices [24]. According to previous results saturated aldehydes (SAs) that are structurally very similar to PUAs are not active and represent ideal controls due to their physicochemical resemblance to the antiproliferative metabolites [25]. We therefore applied a SA derived probe (TAMRA-SA in Figure 1) with otherwise identical properties compared to TAMRA-PUA. These probes proved to be valuable tools to monitor PUA uptake and to answer the question if accumulation takes place in specific organs of the copepods. Indeed, accumulation of TAMRA-PUA was visible in the gonads of *A. tonsa* while TAMRA-SA enriched in the lipid sac.

## Materials and Methods

### Synthesis of the probe and other molecules

A DD derived synthetic probe (TAMRA-PUA) synthesized from 2*E*,4*E*-decadien-9-ynal (DDY) containing the fluorophore 5-tetramethylrhodamine, a hexanal derived probe containing 5-tetramethylrhodamine (TAMRA-SA) and 5-tetramethylrhodamine-azide (TAMRA-N_3_) were synthesized (Figure 1, Information S1).

### Culturing of *O. marina* and incubation with the probes

**Figure 2:**
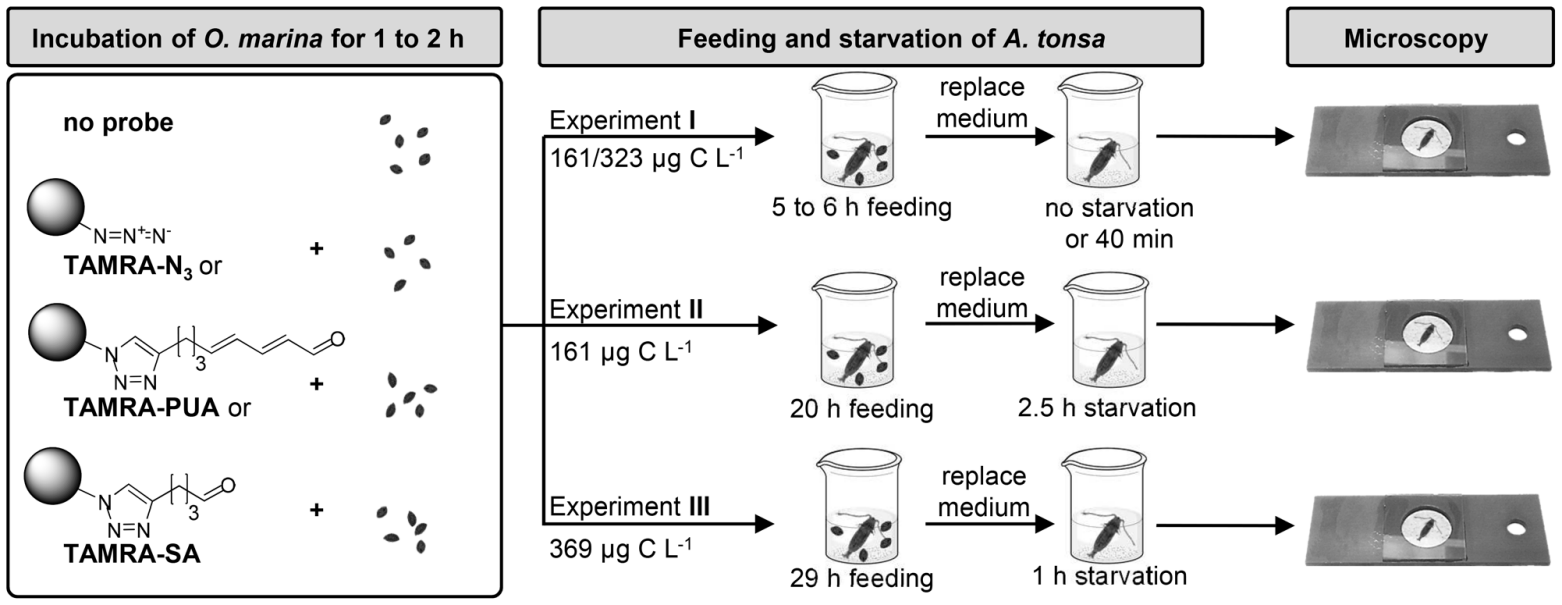
Schematic representation of the experimental set-up. In experiment I incomplete defecation was observed, experiment III was only performed with TAMRA-SA and TAMRA-N_3_.

*Oxyrrhis marina* (Dujardin) CCMP605, obtained from the Provasoli-Guillard National Center for Marine Algae and Microbiota, (NCMA, East Boothbay, Maine, USA), was used as food for copepods and carrier of the molecular probes. Cultures were kept with *Dunaliella tertiolecta* (Butcher) CCMP1320 as food source. Before copepod feeding experiments *O. marina* cultures (9.4×10^3^ cells in 10 mL f/2) were fed with *D. tertiolecta* (500 µL of a 728×10^3^ cells mL^−1^ culture) and kept for six days at 22°C without light in f/2 medium [26]. For experiments with copepods only cultures with low *D. tertiolecta* content (cell number lower than one tenth of *O. marina*) were used. To load *O. marina* with molecular probes, these cultures were incubated in the dark at 22°C for 2 h (experiment **I**) or 1 h (experiments **II**, **III**) with 10 µM TAMRA-PUA, TAMRA-N_3_ or TAMRA-SA, respectively (for structures see Figure 1). Probes were administered as 5 mM stock solutions in DMSO at *O. marina* cell densities of 12.5×10^3^ cells mL^−1^ (**I**, **II**) and 5.0×10^3^ cells mL^−1^ (**III**). A schematic representation of the experiments is given in Figure 2.

### Sampling and selection of *A. tonsa* and feeding with the probe treated *O. marina*

*Acartia tonsa* (Dana) was sampled during daytime between 6–9 April 2013 from the dock of the Skidaway Institute of Oceanography, Savannah, Georgia, USA by towing a 200-µm mesh net perpendicularly from the bottom to surface. The content of the cod end was transported immediately to the laboratory, where adult females were sorted out for the experiments by pipetting under a dissecting microscope. Individual females were kept in 20 mL polystyrene vessels (Sample cup 722060, Dynalon Labware, Rochester, New York, USA used in experiments **I** and **II**) or 2 mL polystyrene well plates (MultiDish 150628, Nalgene Nunc, Penfield, New York, USA used in experiment **III**) filled with filtered dock water (0.7 µm, GF/F Whatman, Clifton, NJ, USA). Females were either kept on un-incubated control food or treated with 10 µM TAMRA-PUA, TAMRA-SA or TAMRA-N_3_ pre-incubated *O. marina* cultures (see above). The final cell density/carbon content of *O. marina* for experiment **I** was adjusted to 313 cells mL^−1^/161 µg C L^−1^ for TAMRA-PUA and TAMRA-SA and 625 cells mL^−1^/323 µg C L^−1^ for TAMRA-N_3_ and controls. For experiment **II** 313 cells mL^−1^/161 µg C L^−1^ and for **III** 714 cells mL^−1^/369 µg C L^−1^ were used. Vessels and wells were kept dark by a cover of aluminium foil to reduce growth of remaining *D. tertiolecta* and incubated in the climate chamber at 22°C for 5 to 6 h (**I**), 20 h (**II**) or 29 h (**III**). The feeding period was ended by replacing the medium containing *O. marina* with filtered dock water four times using a pipette. Thereafter copepods were either sampled immediately (experiment **I** for TAMRA-N_3_ and non-treated algae) or the copepods were left to starve in the prey free dock water for 40 min (experiment **I** for TAMRA-PUA and TAMRA-SA treated copepods) before sampling. All copepods were starved for 2.5 h in experiment **II** or 1 h in experiment **III** before sampling. The copepods were sampled and fixed by placing them in a bottom plate of an Utermöhl sedimentation chamber containing filtered dock water and glutaraldehyde (final concentration 0.3%).

### Fluorescence microscopy

Fluorescence and bright field images were taken with an Olympus IX-50 inverted epifluorescence microscope (Tokyo, Japan) equipped with a 100-W mercury burner and a 1.4 MP colour CCD microscopy camera (M14, by Lumenera, Ottawa, Ontario, Canada). The Olympus filter cube U-MNG (NG) was used with a 530–550 nm excitation filter, a DM 570 nm dichroic mirror, and a BA 590 nm barrier filter. The microscope's magnification is 2.5, either 4x (UPlan Fl 4x) or 10x (UPlan Fl 10x phase) objectives were used to observe copepods. Unless otherwise indicated epifluorescence pictures of *A. tonsa* were taken with 500 ms exposure time.

Bright field images were transferred into monochrome pictures and processed with Adobe Photoshop CS6 regarding tonal correction. For overlays of fluorescence and bright field images, the black channel of each fluorescence image was removed and selective color settings were optimized, whereby the same routine and settings were applied to all images. All unmodified pictures are available in Folder S1.

## Results and Discussion

### Design of the probe and control substances

Previous work showed that the activity of PUAs is highly structure specific. While a series of medium size α,β,γ,δ-unsaturated aldehydes tested showed activity, SAs were completely inactive even at elevated concentrations. Interestingly, activity of α,β,γ,δ-unsaturated aldehydes was not determined by the polarity of the terminus thus offering a potential site for structural manipulation [25], [27]. We therefore developed a probe containing a C10 α,β,γ,δ-unsaturated aldehyde structure element linked at the terminus with the well-established and commercially available fluorophore tetramethylrhodamine (TAMRA) (Figure 1). The approach offers the possibility to localize TAMRA-PUA and other TAMRA containing molecules by fluorescence microscopy without interfering with the active structural element. For control experiments we synthesized a probe with similar structure only modifying the aldehyde head group. By exchanging the α,β,γ,δ-unsaturated aldehyde motive with a saturated aldehyde structure we obtained the probe TAMRA-SA modeled after the inactive structures [27]. The corresponding fluorescent azide (TAMRA-N_3_) was monitored in control experiments to exclude that accumulation due to the structure of the fluorophore itself occurs in the copepods.

### Feeding procedure and defecation of *A. tonsa*

Previous experiments showed that PUAs can be delivered on the food algae *P. minimum* to the copepod *C. helgolandicus*. If this dinoflagellate is incubated in a PUA solution in seawater it adsorbs some of the compounds and delivers them if added as a food source to the copepods [7]. In pilot experiments we loaded 10 µM TAMRA-PUA on *P. minimum* and delivered it to *A. tonsa*. Unfortunately, the strong autofluorescence of *P. minimum* interfered with the detection of the TAMRA fluorescence (see Figure S1). It was thus impossible to distinguish between TAMRA and chlorophyll fluorescence. To overcome this limitation we reasoned that a heterotrophic food source that does not contain any photosynthetic pigments could act as a shuttle of the probe. The heterotrophic dinoflagellate *O. marina* is known to be a good food source for *A. tonsa* (reviewed in [28]) and the first experiments demonstrated that TAMRA-PUA can be loaded on this prey (Figure S2). The dinoflagellate *O. marina* was fed with the diatom *D. tertiolecta*. To avoid interference of the pigments of the prey alga, six days before the onset of feeding experiments, *O. marina* cultures were transferred into the dark to arrest growth of the algae. Remaining algae were removed from the culture by predation of *O. marina*. Before the experiments were conducted, it was verified by microscopy that the *D. tertiolecta* cell count was significantly lower than that of *O. marina*, a criterion indicating sufficient suppression of algal autofluorescence. Before conducting the final experiments, preliminary treatments were performed to determine optimum probe-concentration and incubation times. In the experiments shown, one *O. marina* culture was split in four equal parts and either incubated for 1 h (experiments **II**, **III**) or 2 h (**I**) with 10 µM TAMRA-PUA, 10 µM TAMRA-SA or 10 µM TAMRA-N_3_. The schematic overview of the experiments is presented in Figure 2. One control received no treatment.

Single individuals of *A. tonsa* females were sorted into filtered dock water and pre-treated or control *O. marina* cultures were added to give final cell densities between 313 cells ml^−1^ and 714 cells ml^−1^. After feeding for 5 to 29 h, the *O. marina* containing medium was replaced by filtered dock water. Copepods were either sampled directly or starved for up to 2.5 h before sampling. Immediately after sampling fluorescence microscopy was conducted to reveal the localization of the probes within the animals.

**Figure 3:**
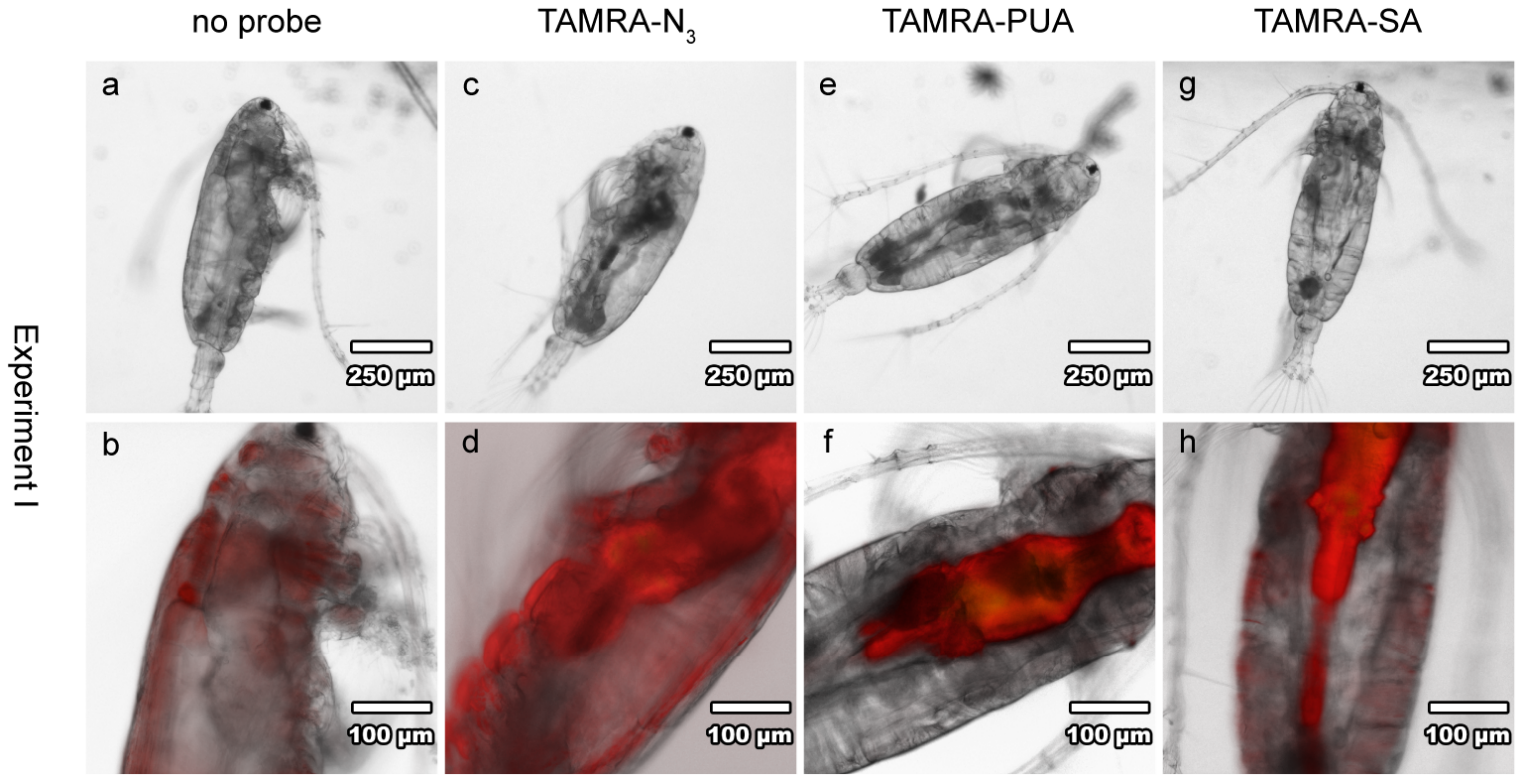
Overview about feeding experiment I with *A. tonsa*. Copepods were fed for 5 h on non-treated (**a**, **b**) *O. marina* or on TAMRA-N_3_ (**c**, **d**) loaded *O. marina* (323 µg C L^−1^) without starvation. Copepods fed for 6 h with TAMRA-PUA (**e**, **f**) or TAMRA-SA (**g**, **h**) pre-treated *O. marina* (161 µg C L^−1^) and starved for 40 min still showed gut fluorescence. First line (**a**, **c**, **e**, **g**): light microscopy images; second line (**b**, **d**, **f**, **h**): overlay light microscopy and epifluorescence images.

**Figure 4:**
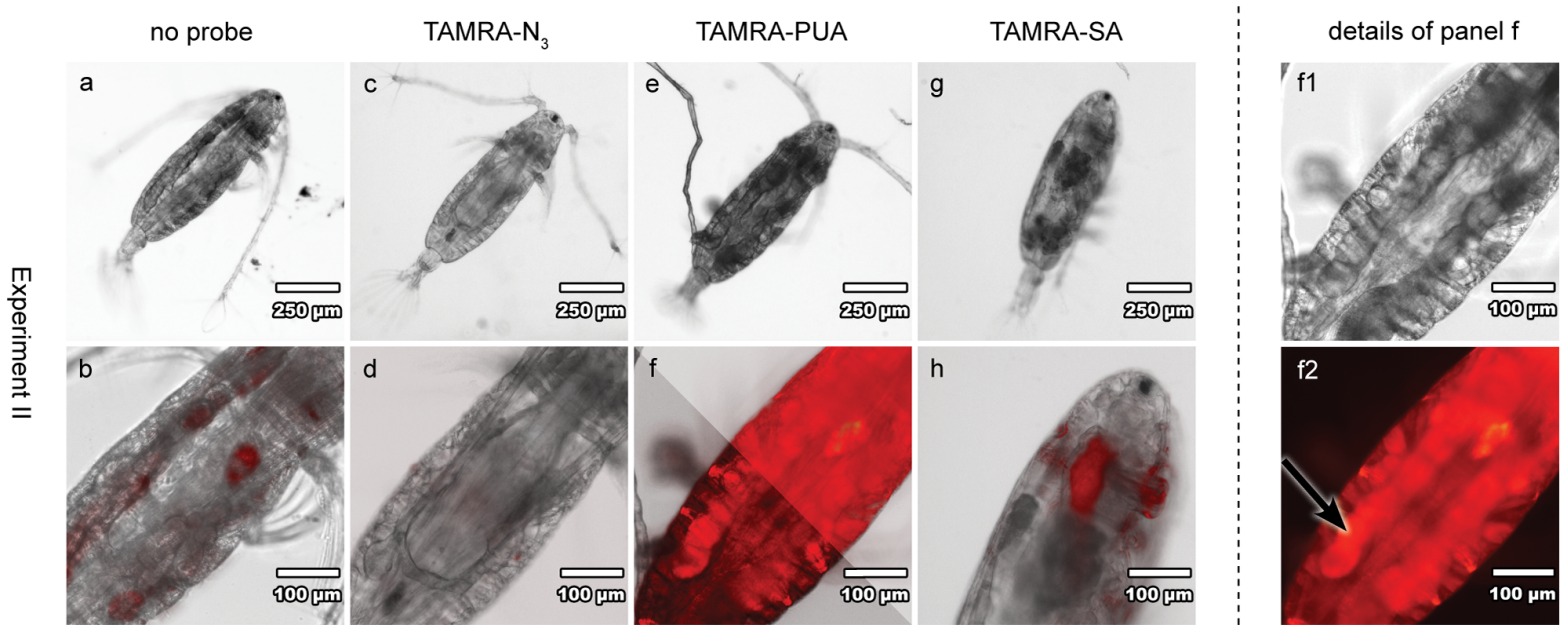
Overview about feeding experiment II with *A. tonsa*. Copepods were fed for 20 h on pre-treated or non-treated *O. marina* (161µg C L^−1^) followed by 2.5 h starvation. Strong fluorescence of the TAMRA-PUA treated copepod (**e**, **f**, **f1**, **f2**) is observed, the arrow indicates high accumulation of TAMRA-PUA in gonad tissue (**f2**). First line (**a**, **c**, **e**, **g**, **f1**): light microscopy images; second line (**b**, **d**, **f**, **h**): overlay light microscopy and epifluorescence images; **f2**: epifluorescence image. Overlay image **f** is bisected – right section: with routine applied to all other fluorescence pictures, left section: changed settings for selective colour correction.

**Figure 5:**
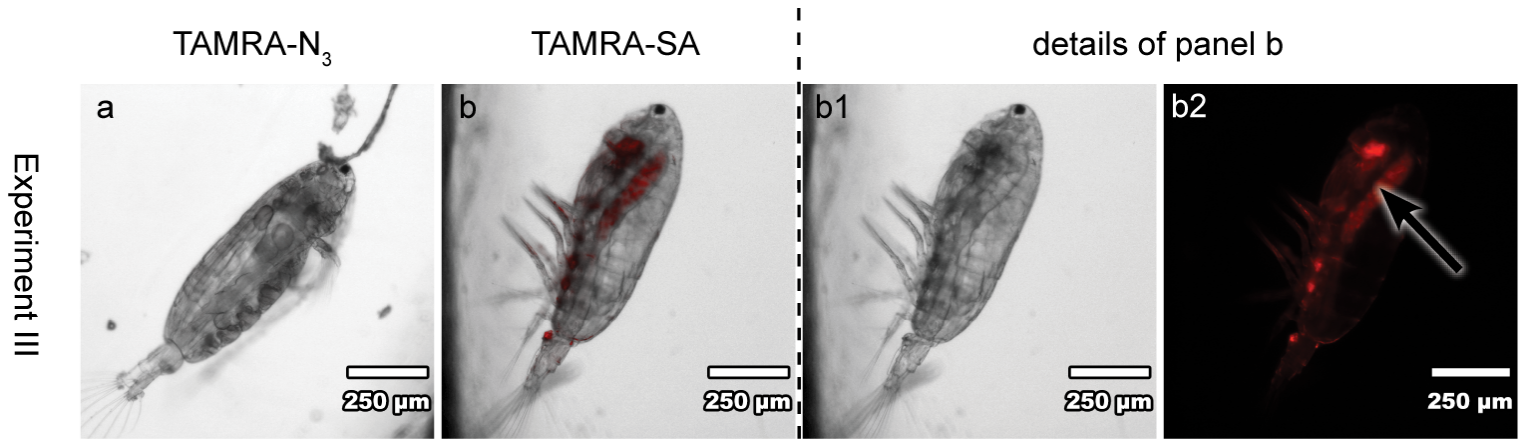
Feeding experiment III with *A. tonsa*. Copepods were fed for 29 h with TAMRA-N_3_ (**a**, exposure time 1.5 s) or TAMARA-SA (**b**, **b1**, **b2** exposure time 500 ms) pre-treated *O. marina* (369 µg C L^−1^) and starved for 1 h. The arrow indicates high accumulation of TAMRA-SA in the lipid sac (**b2**). **a, b**: overlay light microscopy and epifluorescence images; **b1**: light microscopy image; **b2** epifluorescence image.

We first checked for the effect of defecation and washing of copepods (experiment **I**) to minimize fluorescence on the surface of copepods or in the digestive tract that might interfere with a possible detection of TAMRA-accumulation in copepod tissue (Figure 3). Without starvation a high and unspecific fluorescence was observed if copepods were incubated with TAMRA-N_3_ (Figure 3d). This might result from unspecific adsorption of the fluorescent dye to the surface of the copepods. This unspecific fluorescence could be eliminated within a 1 to 2.5 h starvation and washing time in fresh medium (Figure 3, Figure 4 and Figure 5). Also the observed fluorescence in the stomach and gut area of the copepods could be reduced significantly by 1 to 2.5 h starvation (and defecation). Earlier reports indicated reduced fluorescence of photopigments of food algae in *A. tonsa* after 120 min starvation to ca. 5% of initial values [29]. Indeed, only minor TAMRA-N_3_ fluorescence was observed in the gut after this period indicating that the unmodified fluorophore does not behave significantly different compared to food pigments (Figure 4d and Figure 5a). Copepods fed *O. marina* that was not incubated with a fluorophore did not show any signal in the digestive tract. This indicates a successful elimination of autofluorescence of *O. marina* food algae (Figure 4b). Remaining fluorescence in the digestive tract thus originates from TAMRA derivatives and not the autofluorescence of *D. tertiolecta*.

### Uptake and localization of TAMRA-PUA

When copepods were fed for brief periods of 5 to 6 h on TAMRA-PUA and TAMRA-SA pre-treated *O. marina* cells as food source and a starvation of 40 min was allowed (Figure 2), enrichment of fluorescence was mainly detected in the gut (Figure 3f, h). These probes remained longer in the digestive tract in comparison to TAMRA-N_3_. Assuming a clearance of ca. 80% of gut fluorescence from pigmented food algae after 40 min [29], remaining fluorescence would likely be due to residual TAMRA-loaded food. Also the increased hydrophobicity of the aldehyde-probes might be responsible for longer residual times in the gut. However, since we aimed to elucidate the localization of PUAs within the copepod tissue, we chose longer feeding and defecation times in the subsequent experiments. For optimization, we incubated *A. tonsa* under different conditions, e.g. copepod feeding and starvation times as well as food carbon content. A starvation time of at least 1 h usually enabled more complete defecation and offered the possibility to observe areas of probe accumulation without interfering fluorescent gut content.

Most intensive tissue fluorescence in TAMRA-PUA treated copepods was reached with condition **II** (161 µg C L^−1^, 20 h feeding time and 2.5 h starvation) (Figure 4f, f2). With remarkable selectivity, the probe accumulated preferentially in the gonads (Figure 4f2, arrow). This accumulation supports the activity of PUAs as antiproliferative metabolites. The targeted delivery of these reactive metabolites to the reproductive organs explains observation that diatom rich diets cause oocyte degradations characterised by cell fragmentation, presence of apoptotic bodies and degradation of cytoplasm in *Calanus helgolandicus* [9]. In that study histological and cytological observations on gonads and oocyte development stages in *C. helgolandicus* were performed and several mechanisms as well as other factors besides PUA content of the diet were postulated to cause adverse effects in copepod oocyte maturation. A targeted delivery of active metabolites at least partially explains the observed selectivity of adverse effects. Our finding thus provides an explanation for how PUAs can be teratogenic to copepods without any other obvious harmful effect on the feeding adults [30]. Accumulation might be facilitated by covalent reactions of the DD analogue TAMRA-PUA since DD acts as electrophile: PUAs are known to be attacked by nucleophiles, such as amine or thiol groups of proteins [31], [32] or amine groups of DNA [33]–[35]. Such reactions lead to modifications of enzymes that might lead to dysfunctions, functional alterations and thus interference with molecular functions and biological processes. DNA modifications might disturb transcription and translation. In addition, DD enhances oxidative stress which causes DNA breaks [36].

### Uptake and localization of TAMRA-SA

Experimental setup **III** with increased carbon content of 369 µg C L^−1^, increased copepod feeding time of 29 h and 1 h starvation showed an accumulation of TAMRA-SA in the lipid sac of a female (Figure 5b, b2 arrow). TAMRA-SA is the most nonpolar of all tested substances and therefore incorporation in the lipid sac of *A. tonsa* might be caused by its physicochemical properties. This result is in agreement with the fact that copepods store part of their food lipids in a lipid sac for energy supply and reproduction [37]. The results indicate that there are different accumulation behaviours of PUAs and SAs besides the physicochemical similarities of these molecules. PUAs enrich in gonads, where they probably interfere with enzyme activity and physiological functions resulting in their teratogenic activity. In contrast, their saturated non-toxic counterparts seem to be enclosed in the lipid sac for storage.

## Conclusion

In this study we introduce the delivery and use of molecular probes designed to monitor the action of PUAs in copepods. We provide evidence for a targeted localization of a PUA-derived molecular probe in the gonads of *A. tonsa*. These effects could be responsible for PUAs' selective teratogenic action. We also show that a probe derived from an inactive SA accumulates in the lipid bodies thereby further supporting the notion of selectivity of PUAs.

## Supporting Information

Figure S1 **Bright field and epifluorescence images of** ***Prorocentrum minimum*** **.** Algae cells were treated without (**a**, **b**) and with 10 µm TAMRA-PUA after 1 h (**c**, **d**) and 22 h (**e**, **f**) incubation time. For epifluorescence images the exposure time was 405 ms. Cells were measured with an Olympus HX-60 equipped with an U-MSWG filter cube containing an BP 480–550 nm excitation filter, a DM 570 nm dichroic mirror and an BA 590 nm emission filter combined with a Retiga 1300 camera.
(TIF)

Figure S2 **Bright field and epifluorescence images of** ***Oxyrrhis marina*** **.** Cells were treated without (**a**, **b**, **c**) and with 10 µm TAMRA-PUA after 19 h (**d**, **e**, **f**) incubation time. For epifluorescence images the exposure time was 200 ms (**b**, **e**) or 405 ms (**c**, **f**). The cells were measured as described for Figure S1.
(TIF)

Folder S1 **Unmodified light microscopy and epifluorescence images of** ***A. tonsa*** **.**
(ZIP)

Information S1 **Experimental procedures and characterization data of synthetic products.**
(DOCX)
