# Supplementary figures and images for: Accumulation of Polyunsaturated Aldehydes in the Gonads of the Copepod Acartia tonsa Revealed by Tailored Fluorescent Probes
Source: PLoS One. 2014 Nov 10;9(11):e112522. doi: 10.1371/journal.pone.0112522 (PMC4226538; doi:10.1371/journal.pone.0112522)

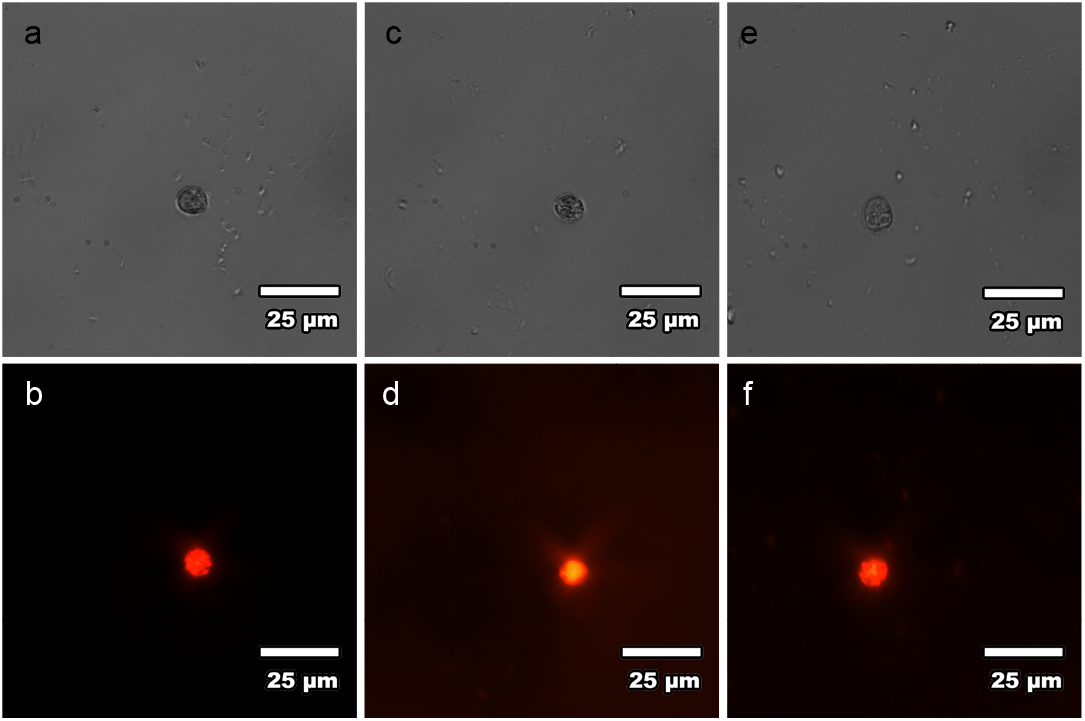

Supplement: Figure S1 — Bright field and epifluorescence images of Prorocentrum minimum . Algae cells were treated without (a, b) and with 10 µm TAMRA-PUA after 1 h (c, d) and 22 h (e, f) incubation time. For epifluorescence images the exposure time was 405 ms. Cells were measured with an Olympus HX-60 equipped with an U-MSWG filter cube containing an BP 480–550 nm excitation filter, a DM 570 nm dichroic mirror and an BA 590 nm emission filter combined with a Retiga 1300 camera. (TIF) [file pone.0112522.s001.tif]

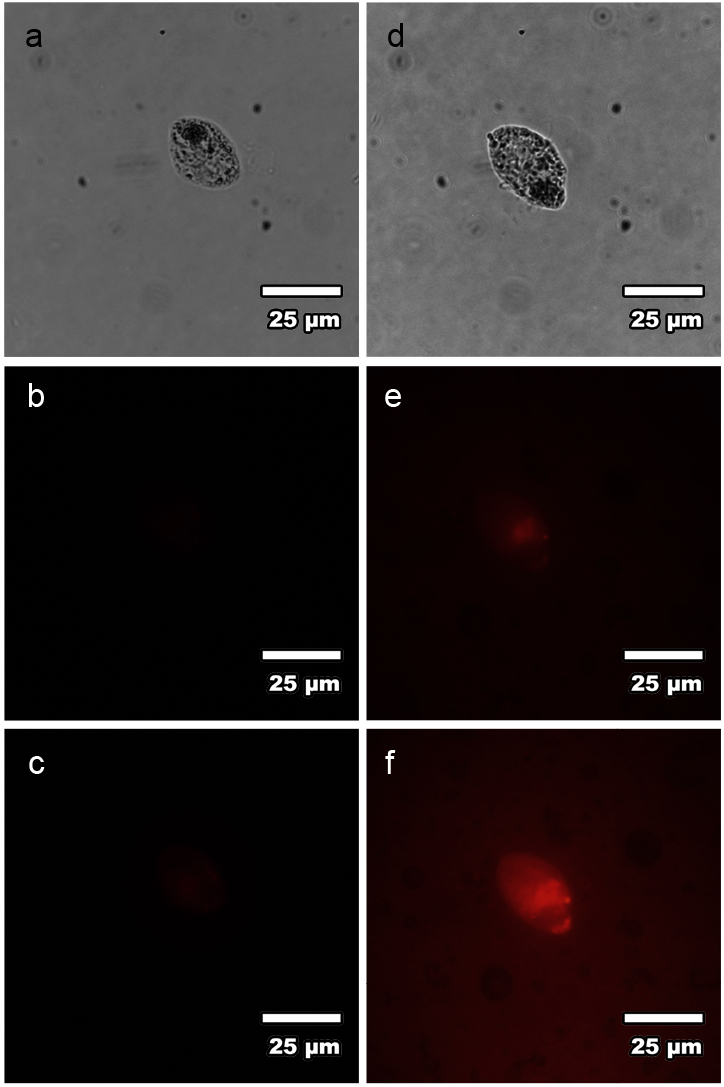

Supplement: Figure S2 — Bright field and epifluorescence images of Oxyrrhis marina . Cells were treated without (a, b, c) and with 10 µm TAMRA-PUA after 19 h (d, e, f) incubation time. For epifluorescence images the exposure time was 200 ms (b, e) or 405 ms (c, f). The cells were measured as described for Figure S1. (TIF) [file pone.0112522.s002.tif]

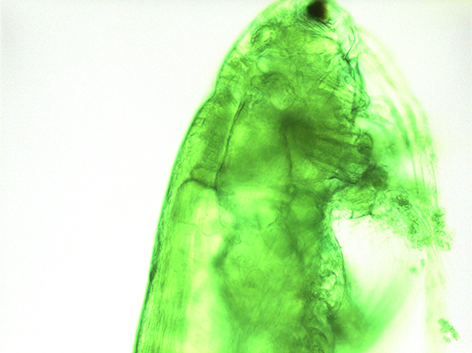

Supplement: Folder S1 — Unmodified light microscopy and epifluorescence images of A. tonsa . (ZIP) [file pone.0112522.s003.zip › images/ExpI no probe 10x bf.tif]

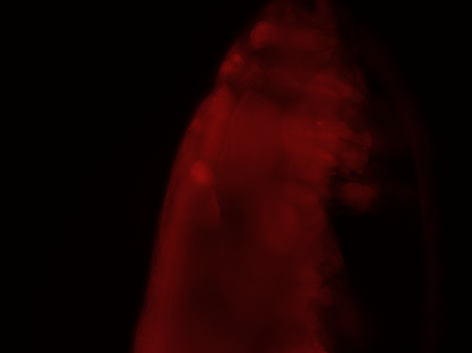

Supplement: Folder S1 — Unmodified light microscopy and epifluorescence images of A. tonsa . (ZIP) [file pone.0112522.s003.zip › images/ExpI no probe 10x fluo.tif]

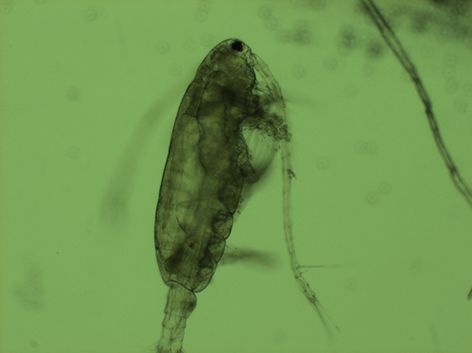

Supplement: Folder S1 — Unmodified light microscopy and epifluorescence images of A. tonsa . (ZIP) [file pone.0112522.s003.zip › images/ExpI no probe 4x bf.tif]

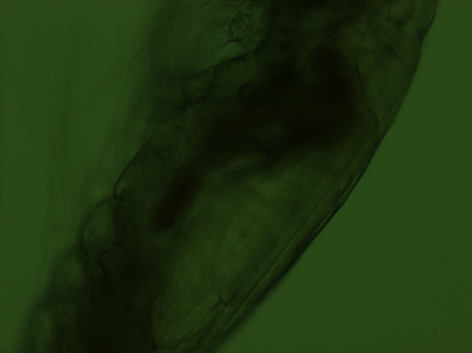

Supplement: Folder S1 — Unmodified light microscopy and epifluorescence images of A. tonsa . (ZIP) [file pone.0112522.s003.zip › images/ExpI TAMRA-N3 10x bf.tif]

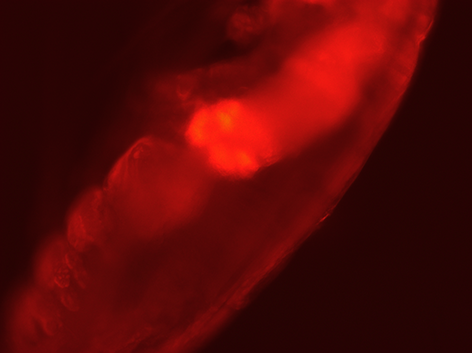

Supplement: Folder S1 — Unmodified light microscopy and epifluorescence images of A. tonsa . (ZIP) [file pone.0112522.s003.zip › images/ExpI TAMRA-N3 10x fluo.tif]

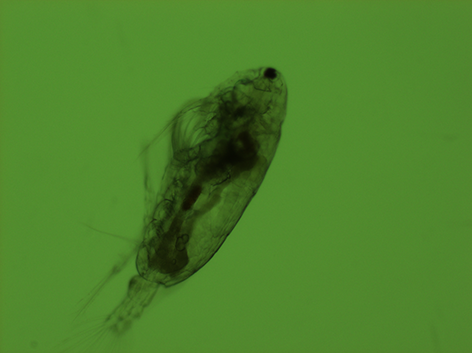

Supplement: Folder S1 — Unmodified light microscopy and epifluorescence images of A. tonsa . (ZIP) [file pone.0112522.s003.zip › images/ExpI TAMRA-N3 4x bf.tif]

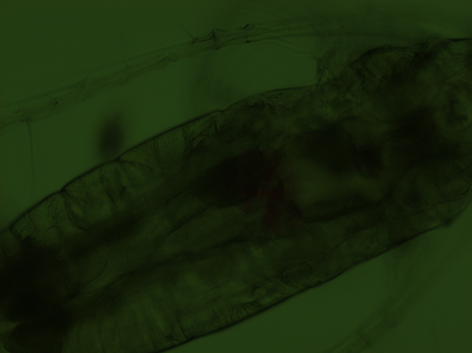

Supplement: Folder S1 — Unmodified light microscopy and epifluorescence images of A. tonsa . (ZIP) [file pone.0112522.s003.zip › images/ExpI TAMRA-PUA 10x bf.tif]

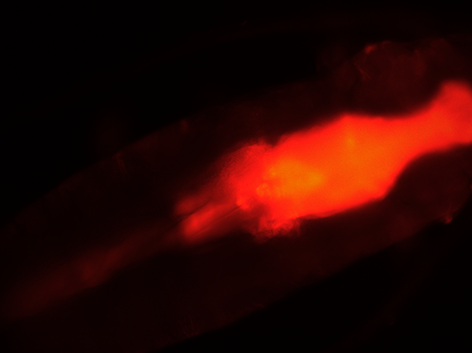

Supplement: Folder S1 — Unmodified light microscopy and epifluorescence images of A. tonsa . (ZIP) [file pone.0112522.s003.zip › images/ExpI TAMRA-PUA 10x fluo.tif]

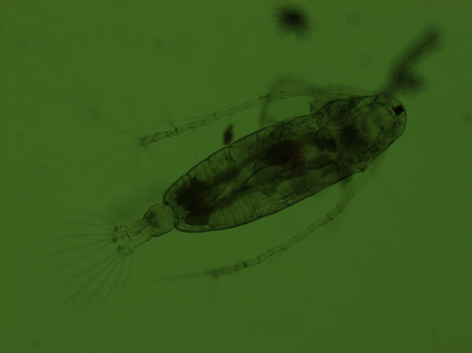

Supplement: Folder S1 — Unmodified light microscopy and epifluorescence images of A. tonsa . (ZIP) [file pone.0112522.s003.zip › images/ExpI TAMRA-PUA 4x bf.tif]

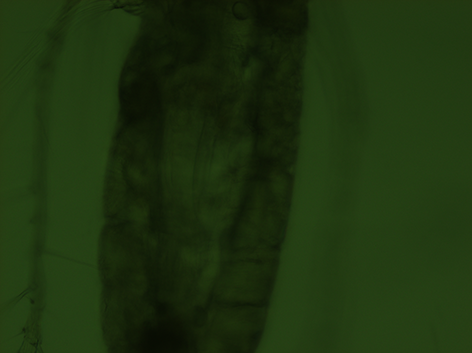

Supplement: Folder S1 — Unmodified light microscopy and epifluorescence images of A. tonsa . (ZIP) [file pone.0112522.s003.zip › images/ExpI TAMRA-SA 10x bf.tif]

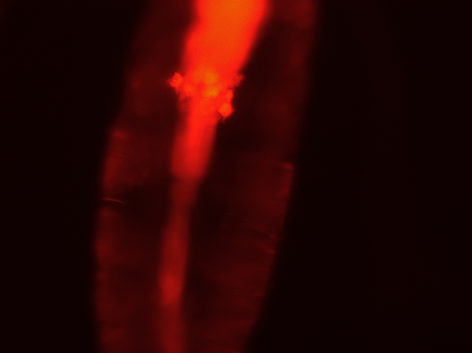

Supplement: Folder S1 — Unmodified light microscopy and epifluorescence images of A. tonsa . (ZIP) [file pone.0112522.s003.zip › images/ExpI TAMRA-SA 10x fluo.tif]

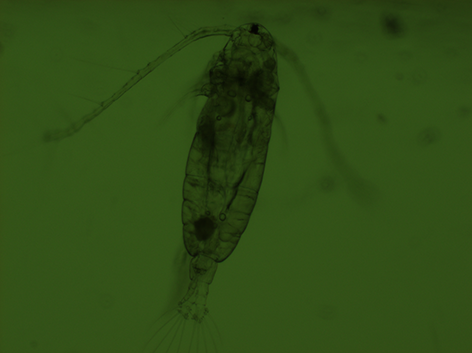

Supplement: Folder S1 — Unmodified light microscopy and epifluorescence images of A. tonsa . (ZIP) [file pone.0112522.s003.zip › images/ExpI TAMRA-SA 4x bf.tif]

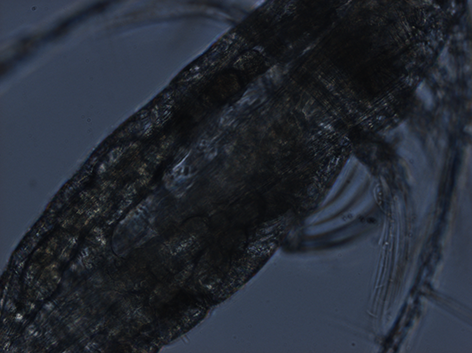

Supplement: Folder S1 — Unmodified light microscopy and epifluorescence images of A. tonsa . (ZIP) [file pone.0112522.s003.zip › images/ExpII no probe 10x bf.tif]

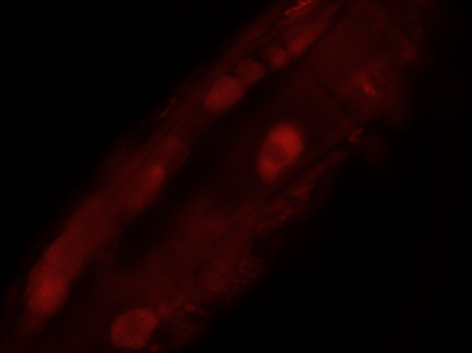

Supplement: Folder S1 — Unmodified light microscopy and epifluorescence images of A. tonsa . (ZIP) [file pone.0112522.s003.zip › images/ExpII no probe 10x fluo.tif]

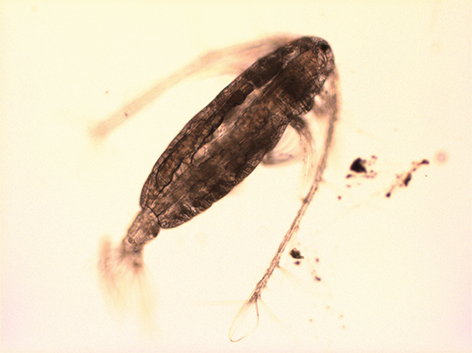

Supplement: Folder S1 — Unmodified light microscopy and epifluorescence images of A. tonsa . (ZIP) [file pone.0112522.s003.zip › images/ExpII no probe 4x bf.tif]

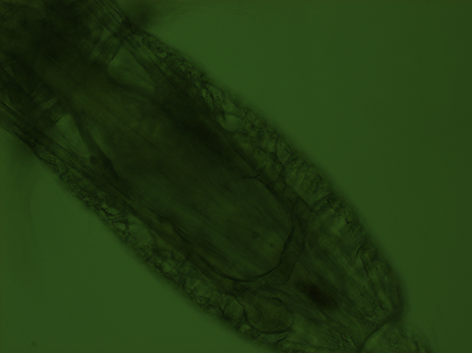

Supplement: Folder S1 — Unmodified light microscopy and epifluorescence images of A. tonsa . (ZIP) [file pone.0112522.s003.zip › images/ExpII TAMRA-N3 10x bf.tif]

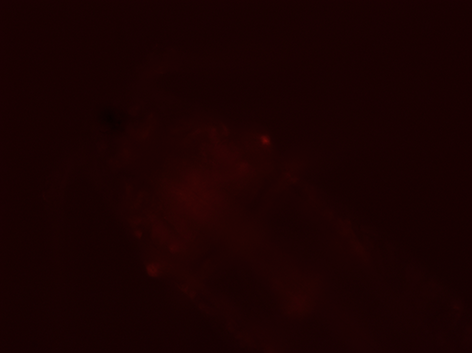

Supplement: Folder S1 — Unmodified light microscopy and epifluorescence images of A. tonsa . (ZIP) [file pone.0112522.s003.zip › images/ExpII TAMRA-N3 10x fluo.tif]

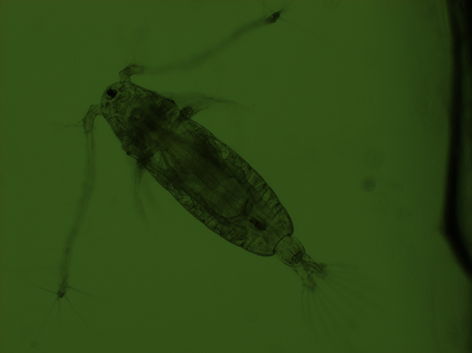

Supplement: Folder S1 — Unmodified light microscopy and epifluorescence images of A. tonsa . (ZIP) [file pone.0112522.s003.zip › images/ExpII TAMRA-N3 4x bf.tif]

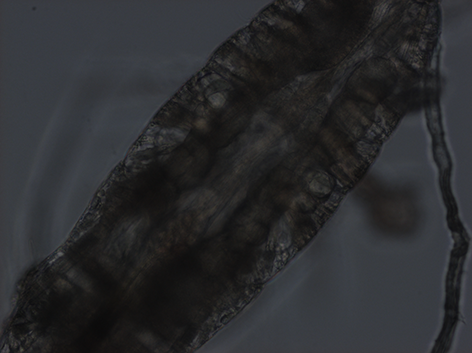

Supplement: Folder S1 — Unmodified light microscopy and epifluorescence images of A. tonsa . (ZIP) [file pone.0112522.s003.zip › images/ExpII TAMRA-PUA 10x bf.tif]

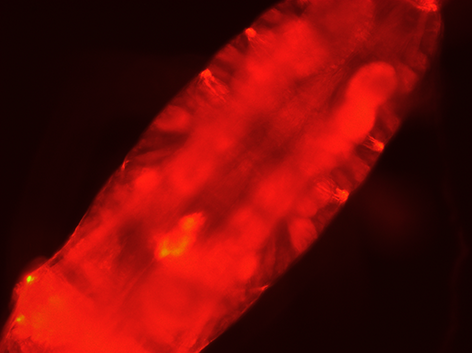

Supplement: Folder S1 — Unmodified light microscopy and epifluorescence images of A. tonsa . (ZIP) [file pone.0112522.s003.zip › images/ExpII TAMRA-PUA 10x fluo.tif]

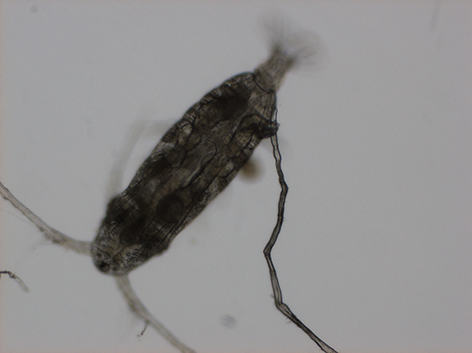

Supplement: Folder S1 — Unmodified light microscopy and epifluorescence images of A. tonsa . (ZIP) [file pone.0112522.s003.zip › images/ExpII TAMRA-PUA 4x bf.tif]

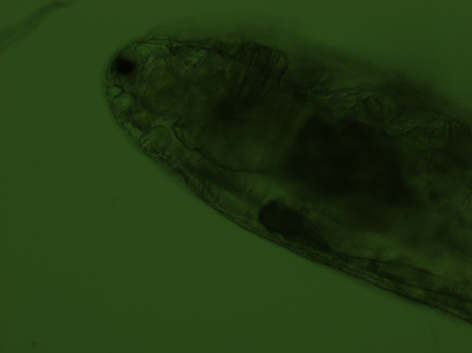

Supplement: Folder S1 — Unmodified light microscopy and epifluorescence images of A. tonsa . (ZIP) [file pone.0112522.s003.zip › images/ExpII TARMA-SA 10x bf.tif]

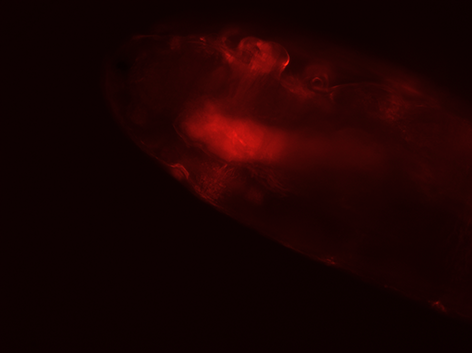

Supplement: Folder S1 — Unmodified light microscopy and epifluorescence images of A. tonsa . (ZIP) [file pone.0112522.s003.zip › images/ExpII TARMA-SA 10x fluo.tif]

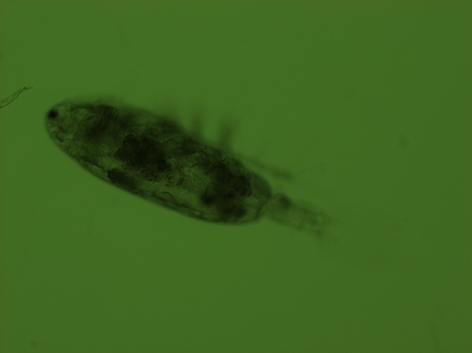

Supplement: Folder S1 — Unmodified light microscopy and epifluorescence images of A. tonsa . (ZIP) [file pone.0112522.s003.zip › images/ExpII TARMA-SA 4x bf.tif]

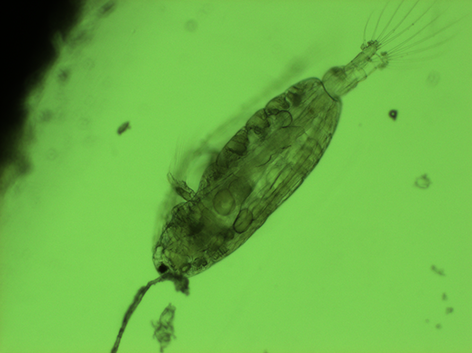

Supplement: Folder S1 — Unmodified light microscopy and epifluorescence images of A. tonsa . (ZIP) [file pone.0112522.s003.zip › images/ExpIII TAMRA-N3 4x bf.tif]

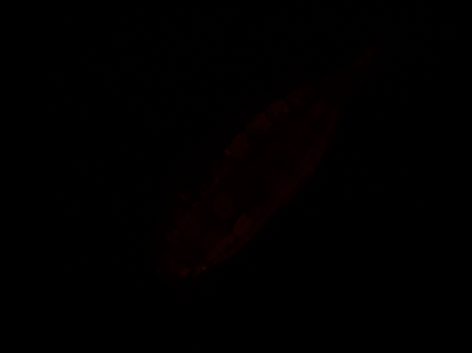

Supplement: Folder S1 — Unmodified light microscopy and epifluorescence images of A. tonsa . (ZIP) [file pone.0112522.s003.zip › images/ExpIII TAMRA-N3 4x fluo 1.5s.tif]

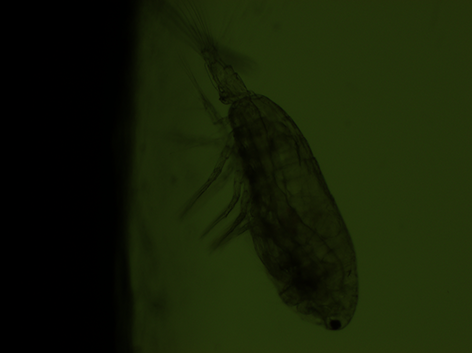

Supplement: Folder S1 — Unmodified light microscopy and epifluorescence images of A. tonsa . (ZIP) [file pone.0112522.s003.zip › images/ExpIII TAMRA-SA 4x bf.tif]

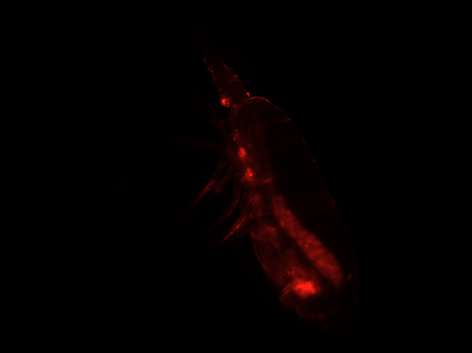

Supplement: Folder S1 — Unmodified light microscopy and epifluorescence images of A. tonsa . (ZIP) [file pone.0112522.s003.zip › images/ExpIII TAMRA-SA 4x fluo.tif]
