# Supplementary material for: Accumulation of Polyunsaturated Aldehydes in the Gonads of the Copepod Acartia tonsa Revealed by Tailored Fluorescent Probes
Source: PLoS One. 2014 Nov 10;9(11):e112522. doi: 10.1371/journal.pone.0112522 (PMC4226538; doi:10.1371/journal.pone.0112522)
Supplement: Information S1 — Experimental procedures and characterization data of synthetic products. (DOCX) [file pone.0112522.s004.docx]

# Supporting Information S1: Experimental procedures and characterization data of synthetic products.

# Accumulation of polyunsaturated aldehydes in the gonads of the copepod *Acartia tonsa* revealed by tailored fluorescent probes

**Stefanie Wolfram^1^, Jens C. Nejstgaard^2,3^ and Georg Pohnert^1^**

**1** Institute for Inorganic and Analytical Chemistry, Friedrich Schiller University, Lessingstr. 8, 07743 Jena, Germany,

**2** Skidaway Institute of Oceanography, 10 Ocean Science Circle, Savannah, GA 31411, USA,

**3** Department of Experimental Limnology, Leibniz-Institute of Freshwater Ecology and Inland Fisheries (IGB), Department 3 Experimental Limnology, Alte Fischerhütte 2, 16775 Stechlin, Germany

# Synthetic procedures

## General remarks

All chemicals were purchased as reagent grade or better and used without further purification. If necessary, reactions were performed under argon. Commercially available dry solvents were employed. Diethyl ether and tetrahydrofuran (THF) contained butylhydroxytoluene as peroxidation inhibitor. Column chromatography was carried out on Merck silica gel (0.04 – 0.063 mesh). Thin layer chromatography (TLC) was performed with TLC silica gel 60 F_254_ plates from Merck. TLC spots were visualized by irradiation of the TLC plate with UV radiation (254 nm) or by dipping in Seebach reagent (2.5 g phosphomolybdic acid and 1 g cer(IV)sulfate dissolved in 65 ml water, slowly acidified by dropwise addition of 6 ml concentrated sulfuric acid). Preparative HPLC was performed with a Shimadzu LC-8A system using a SPD-10AV UV/VIS detector and a LiChro CART® 250-10 Purospher® column (RP C18, endcapped, 5 µm particle size) with solvent A, (water, containing 2 % methanol (v/v)) and solvent B (methanol) using the following program: 0.1 min, 98% A; within 4.9 min to 30% A; within 15.0 min to 0% A; for 7 min, 0% A; within 1 min to 98% A; for 10 min 98% A; flow 4 mL min^-1^.

## 5-Iodo-penta-2*E*,4*E/Z*-dienal

|  |  |
| --- | --- |
| isomer a | isomer b |

5-Iodo-penta-2*E*,4*E/Z*-dienal was synthesized according to Soullez et *al.* as isomeric mixture with a 4*E*:4*Z* ratio of 52:48; spectroscopic data are identical as described in the literature [1].

isomer a

**^1^H-NMR** (600 MHz, CDCl_3_) δ (ppm) = 6.15 (dd; ^3^J = 15.41 Hz; ^3^J = 7.70 Hz; 1H; H-C2); 6.97 (dd; ^3^J = 15.41 Hz; ^3^J = 11.00 Hz; 1H; H-C3); 7.16 (d; ^3^J = 14.31; 1H; H-C5); 7.32 (dd; ^3^J = 14.30 Hz; ^3^J = 11.00 Hz; 1H; H-C4); 9.58 (d; ^3^J = 7.70; 1H; H-C1)

**^13^C-NMR** (50 MHz, CDCl_3_) δ (ppm) = 92.0 (C5); 131.1 (C2); 143.2 (C4); 149.5 (C3); 193.2 (C1)

**GC/EI-MS** *m/z* (relative intensity) = 51 (31); 52 (32); 53 (33); 81 (100); 82 (13); 127 (19); 179 (7); 208 (12)

**HR-EI-TOF-MS** *m/z* = 207.9389 (M^+●^); calculated (C_5_H_5_IO) m/z = 207.9385

isomer b

**^1^H-NMR** (600 MHz, CDCl_3_) δ (ppm) = 6.37 (dd; ^3^J = 15.41 Hz; ^3^J = 8.25 Hz; 1H; H-C2); 7.01-7.05 (m; 2H; H-C4+H-C5); 7.23-7.29 (m; 1H; H-C3); 9.70 (d; ^3^J = 8.25; 1H; H-C1)

**^13^C-NMR** (50 MHz, CDCl_3_) δ (ppm) = 95.0 (C5); 134.9 (C2); 136.6 (C4); 149.7 (C3); 193.5 (C1)

**GC/EI-MS** *m/z* (relative intensity) = 51 (30); 52 (29); 53 (29); 81 (100); 82 (13); 127 (18); 179 (5); 209 (4)

**HR-EI-TOF-MS** *m/z* = 207.9393 (M^+●^); calculated (C_5_H_5_IO) m/z = 207.9385

## 5-Trimethylsilylpent-4-yne-magnesiumchloride

3.2 equ. pelleted magnesium were etched with 4% hydrochloric acid, rinsed with deionized water to neutral pH, rinsed with acetone several times and dried in vaccum/argon using the Schlenk technique. The magnesium was baked out in vacuum using a heat gun. In an argon atmosphere THF (0.1 mL per 1 mM magensium) and a solution of 1.0 equ. of 5-chloro-1-trimethylsilylpent-1-yne in THF (1.0 mL per 1 mM 5-chloro-1-trimethylsilylpent-1-yne) were added. Grignard reaction was initiated by dropwise adding a maximum of 2 equ. 1,2-dibromoethane. The suspension was refluxed for 2-4 hours with stirring; conversion was checked by GC-MS. The reaction mixture was used *in situ* for metal exchange to trimethylsilylpent-4-yne-zinc(II)bromide on the same day.

## 5-Trimethylsilylpent-4-yne-zinc(II)bromide

Trimethylsilylpent-4-yne-zinc(II)bromide was synthesized according to Adolph et *al.* [2]. The suspension was allowed to settle down before further transformation of the supernatant on the same day.

## (2*E*,4*E*)-10-(Trimethylsilyl)decadien-9-ynal

In an argon atmosphere 0.06-0.07 equ. tetrakis(triphenylphosphine)palladium(0) were added to 1.0 equ. of the mixture of isomers 5-Iodo-penta-2*E*,4*E/Z*-dienal in THF (1 mL per 0.1 mmol). 3.1 to 5.0 equ. of the clear solution of 5-trimethylsilylpent-4-yne-zinc(II)bromide were added. The reaction was hydrolyzed after 5 to 10 min and extracted 3 times with diethyl ether. The combined organic phases were washed with brine and dried with MgSO_4_. Column chromatography was utilized with petrol ether/diethyl ether (4/1, v/v). A light yellow oil was obtained with 28 to 30% yield; it contained approximately 5% of the *2E*,4*Z* isomer analyzed by GC-MS.

**^1^H-NMR** (400 MHz, CDCl_3_) δ (ppm) = 0.14 (s; 9H; SiC(CH_3_)_3_); 1;65 (quin; ^3^J = 7.32 Hz; 2H; H-C7); 2.26 (t; ^3^J = 6.95 Hz; 2H; H-C8); 2.33 (m; 2H; H-C6); 6.08 (dd; ^3^J = 15.00 Hz; ^3^J = 7.68 Hz; 1H; H-C2); 6.25 (dt; ^3^J = 15.00 Hz; ^3^J = 6.59 Hz; 1H; H-C5); 6.34 (dd; ^3^J = 15.37 Hz; ^3^J = 10.25 Hz; 1H; H-C4); 7.07 (dd; ^3^J = 15.37 Hz; ^3^J = 10.25 Hz; 1H; H-C3); 9.53 (d; ^3^J = 8.05 Hz; 1H; H-C1)

**^13^C-NMR** (100 MHz, CDCl_3_) δ (ppm) = 0.11 (SiC(CH_3_)_3_); 19.3 (C8); 27.3 (C7); 32.0 (C6); 85.4 (C9); 106.3 (C10); 129.3 (C4); 130.4 (C2); 145.7 (C5); 152.3 (C3); 193.8 (C1)

**GC/EI-MS** *m/z* (relative intensity) = 67 (7); 73 (100);74 (10); 75 (27); 79 (13); 81 (27); 91 (21); 96 (7); 109 (7); 115 (12); 116 (11); 117 (10); 128 (7); 129 (21); 130 (10); 131 (34); 147 (8); 192 (7);205 (7); 219 (7)

**HR-EI-TOF-MS** *m/z* = 205.1051 ([M-CH_3_]^+^); calculated (C_12_H_17_OSi) *m/z* = 205.1049; (no radical cation of entire substance visible)

## 2*E*,4*E*-Decadien-9-ynal

20 mg (0.091 mmol) (2*E*,4*E*)-10-(trimethylsilyl)decadien-9-ynal were dissolved in 4 mL THF and cooled to 0°C. After adding 0.11 mL (0.11 mmol) of a 1.0 M tetra-n-butylammoniumfluoride solution the solution was stirred till complete turnover (verified by TLC) followed by hydrolyzation and extraction (3 times with diethyl ether). The combined organic phases were washed with brine and dried with MgSO_4_. Column chromatography was utilized with petrol ether/diethyl ether (5/1, v/v) and a light yellow oil was obtained with 30% yield. The product contains approximately 5% of the 2*E*,4*Z* isomer (analyzed by GC-MS).

**^1^H-NMR** (400 MHz, CDCl_3_) δ (ppm) = 1.70 (quin; ^3^J = 7.32 Hz; 2H; H-C7); 1.98 (t; ^4^J = 2.56 Hz; 1H; H-C10); 2.24 (td; ^3^J = 6.95 Hz; ^3^J = 2.56 Hz; 2H; H-C8); 2.36 (m; 2H; H-C6); 6.09 (dd; ^3^J = 15.37 Hz; ^3^J = 7.68 Hz; 1H; H-C2); 6.25 (dt; ^3^J = 15.00 Hz; ^3^J = 6.95 Hz; 1H; H-C5); 6.36 (dd; ^3^J = 15.00 Hz; ^3^J = 10.61 Hz; 1H; H-C4); 7.08 (dd; ^3^J = 15.37 Hz; ^3^J = 10.61 Hz; 1H; H-C3); 9.54 (d; ^3^J = 8.05 Hz; 1H; H-C1)

**^13^C-NMR** (100 MHz, CDCl_3_) δ (ppm) = 17.9 (C8); 27.3 (C7); 31.9 (C6); 69.0 (C10); 83.6 (C9); 129.4 (C4); 130.5 (C2); 145.4 (C5); 152.3 (C3); 193.8 (C1)

**GC/EI-MS** *m/z* (relative intensity) = 63 (16); 65 (19); 66 (12); 67 (14); 77 (33); 78 (28); 79 (61); 80 (26); 81 (100); 91 (85); 92 (15); 107 (13); 108 (23); 115 (14); 117 (21); 119 (34)

**HR-EI-TOF-MS** 148.0890 (M^+●^); calculated (C_10_H_12_O): 148.0888

## 5-Hexynal

5-Hexynal was synthesized from 5-hexynol according to the general protocol for Swern oxidation [3] and purified by rectification. A colorless oil was obtained with 50% yield. The spectroscopic data are identical to the literature [4].

**^1^H-NMR** (400 MHz, CDCl_3_) δ (ppm) = 1.85 (quin; ^3^J = 6.95 Hz; 2H; H-C3); 1.98 (t; ^4^J = 2.38 Hz; 1H; H-C6); 2.27 (td; ^3^J = 7.20 Hz; ^4^J = 2.56 Hz; 2H; H-C4); 2.61 (t; ^3^J = 6.83 Hz; H-C2); 9.81 (s; 1H; H-C1)

**^13^C-NMR** (100 MHz, CDCl_3_) δ (ppm) = 17.8 (C4); 20.8 (C3); 42.5 (C2); 69.3 (C6); 83.1 (C5); 201.7 (C1)

## 5-[3-Azidopropylcarbamoyl]-2-[6-(dimethylamino)-3-(dimethylimino)-3*H*-xanthene-9-yl]benzoate (TAMRA-N_3_)

**TAMRA-N_3_** was synthesized according to Richter et al.; spectroscopic data are identical to the literature [5].

**^1^H-NMR** (600 MHz, CD_3_OD) δ (ppm) = 1.95 (quin; ^3^J = 6.60 Hz; 2H; H-C27); 3.28 (s; 12H; H-C21+H-C22+H-C23+H-C24); 3.47 (t; ^3^J = 6.60 Hz; 2H; H-C28); 3.54 (t; ^3^J = 6.60 Hz; 2H; H-C26); 6.91 (d; ^4^J = 2.20 Hz; 2H; H-C13+H-C16); 7.02 (dd; ^3^J = 9.90 Hz; ^4^J = 2.20 Hz; 2H; H-C11+ H-C18); 7.23 (d; ^3^J = 9.35 Hz; 2H; H-C10+H-C19); 7.39 (d; ^3^J = 8.25 Hz; 1H; H-C7); 8.08 (dd; ^3^J = 7.70 Hz; ^4^J = 2.20 Hz; 1H; H-C6); 8.56 (d; ^4^J = 2.20 Hz; 1H; H-C4)

**LC-MS** (ESI positive mode) m/z = 513 [M+H]^+^, 535 [M+Na]^+^

## 2-(6-(Dimethylamino)-3-(dimethyliminio)-3*H*-xanthene-9-yl)-5-(3-(4-((4*E*,6*E*)-8-xoxoocta-4,6-dienyl)-1*H*-1,2,3-triazol-1-yl)propylcarbamoyl)benzoate (TAMRA-PUA)

5.3 mg (0.036 mmol) 2*E*,4*E*-decadien-9-ynal and 12.8 mg (0.025 mmol) **TAMRA-N_3_** were mixed with 6.0 mg (0.011 mmol) tris[(1-benzyl-1*H*-1,2,3-triazol-4-yl)methyl]amine) (TBTA), 4.7 mL phosphate buffered saline, 0.3 mL *tert*-butanol and 6 mL DMSO. 32 µL of a 1.0 M sodium ascorbate solution (0.032 mmol) and 21 µL of a 0.30 M copper sulfate solution (0.006 mmol) were added with stirring. After 8 hours the mixture was loaded on a C18 cartridge (Chromabond®, C18, endcapped, MACHEREY-NAGEL) for solid phase extraction. The column was washed with water and eluted with methanol. The crude product was diluted with deionized water and purified via preparative HPLC. The solvent was removed under reduced pressure yielding in 11.3 mg (0.017 mmol, 68 %) dark red crystals.

**^1^H-NMR** (600 MHz, CD_3_OD) δ (ppm) = 1.86 (quin; ^3^J = 7.30 Hz; 2H; H-C32); 2.28 (m; 2H; H-C33); 2.31 (quin; ^3^J = 6.60 Hz; 2H; H-C27); 2.74 (t; ^3^J = 7.70 Hz; 2H; H-C31); 3.29 (s; 12H; H-C21+H-C22+H-C23+H-C24); 3.51 (t; ^3^J = 6.60 Hz; 2H; H-C26); 4.52 (t; ^3^J = 6.60 Hz; 2H; H-C28); 6.08 (dd; ^3^J = 15.41 Hz; ^3^J = 8.25 Hz; 1H; H-C37); 6.38-6.46 (m; 2H; H-C34+H-C35); 6.93 (d; ^4^J = 2.20 Hz; 2H; H-C13+H-C16); 7.02 (dd; ^3^J = 9.90 Hz; ^4^J = 2.20 Hz; 2H; H-C11+H-C18); 7.24 (d; ^3^J = 9.35 Hz; 2H; H-C10+ H-C19); 7.27 (dd; ^3^J = 15.41 Hz; ^3^J = 9.35 Hz; 1H; H-C36); 7.37 (d; ^3^J = 7.70 Hz; 1H; H-C7); 7.86 (s; 1H; H-C29); 8.05 (d; ^3^J = 7.70 Hz; 1H; H-C6); 8.54 (s; 1H; H-C4); 9.48 (d; ^3^J = 7.70 Hz; H-C38)

**LC-MS** (ESI positive mode) m/z = 331 [M+2H]^2+^; 661 [M+H]^+^; 683 [M+Na]^+^

**2-(6-(Dimethylamino)-3-(dimethyliminio)-3*H*-xanthene-9-yl)-5-(3-(4-(4-oxobutyl)-1*H*-1,2,3-triazol-1-yl)propylcarbamoyl)benzoate (TAMRA-SA)**

5.0 mg (0.052 mmol) 5-hexynal and 11.1 mg (0.022 mmol) **TAMRA-N_3_** were mixed with 3.0 mg (0.006 mmol) TBTA, 5 mL *tert*-butanol, 1 mL DMSO and 5 mL deionized water. 132 µL of a 1.0 M sodium ascorbate solution (0.132 mmol) and 126 µL of a 0.050 M copper sulfate solution (0.006 mmol) were added with stirring. After stirring over night the solvent was removed under reduced pressure, the residue was partly dissolved in 1 mL methanol and 9 ml deionized water and purified via preparative HPLC, the remaining residue mainly contained TBTA and was discarded. The solvent was removed under reduced pressure yielding in 10.2 mg (0.017 mmol, 77 %) dark red crystals.

**^1^H-NMR** (600 MHz, CD_3_OD) δ (ppm) = 1.55-1.67 (m; 2H); 1.72-1.79 (m; 2H); 2.27 (quin; ^3^J = 6.64 Hz; 2H); 2.71 (t; ^3^J = 7.14 Hz; 2H); 3.27 (s; 12H); 3.49 (t; ^3^J = 6.99 Hz; 2H); 4.48-4.53 (m; 3H); 6.91 (d; ^4^J = 2.35 Hz; 2H); 7.01 (dd; ^3^J = 9.77 Hz; ^4^J = 2.67 Hz; 2H); 7.24 (d; ^3^J = 10.04 Hz; 2H); 7.35 (d; ^3^J = 7.58; 1H); 7.83 (s; 1H); 8.06 (dd; ^3^J = 7.86 Hz; ^4^J = 1.85; 1H); 8.52 (d; ^4^J = 1.64; 1H)

**LC-MS** (ESI positive mode) m/z = 305 [M+2H]^2+^; 609 [M+H]^+^; 631 [M+Na]^+^

# References

1. Soullez D, Ple G, Duhamel L (1997) *ω*-Halogeno polyenals: preparation and application to a one-pot synthesis of polyenals from carbonyl compounds. J Chem Soc, Perkin Trans 1: 1639–1646.

2. Adolph S, Poulet SA, Pohnert G (2003) Synthesis and biological activity of α,β,γ,δ-unsaturated aldehydes from diatoms. Tetrahedron 59: 3003–3008.

3. Schwetlick K (2009) Organikum. Weinheim: Wiley-VCH.

4. Amoroso JW, Borketey LS, Prasad G, Schnarr NA (2010) Direct acylation of carrier proteins with functionalized β-lactones. Organic Letters 12: 2330-2333.

5. Richter P, Weißflog J, Wielsch N, Svatoš A, Pohnert G (2013) Functionalized bis-enol acetates as specific molecular probes for esterases. ChemBioChem 14: 2435-2438.
